# Autism Traits and Cognitive Performance: Mediating Roles of Sleep Disturbance, Anxiety and Depression

**DOI:** 10.1007/s10803-022-05742-5

**Published:** 2022-09-22

**Authors:** Gaynor E. McArthur, Eunro Lee, Robin Laycock

**Affiliations:** School of Health and Biomedical Sciences, RMIT University, Plenty Rd, Bundoora, VIC Australia

**Keywords:** Autism traits, Anxiety, Sleep disturbance, Executive function, Theory of mind, Weak central coherence

## Abstract

Theories about autism spectrum disorder (ASD) have addressed cognitive deficits however few have examined how comorbid diagnoses, including sleep disturbance, anxiety and depression contribute to the underlying deficits. We investigated potential mediations of common ASD comorbidities in the relationship between sub-clinical autism traits and cognitive performance using an international community sample. Cognitive tasks assessed working memory [executive functioning (EF) theory], mental state attribution [theory of mind (ToM)], and global/local visual processing [weak central coherence (WCC) theory]. Structural equation modelling (SEM) demonstrated sleep disturbance and anxiety mediated the relationship of autism traits on measures of EF, but not WCC and ToM. This suggests that treating the symptoms of sleep disturbance and anxiety may lead to improvements in working memory.

Autism spectrum disorder (ASD) is a life-long, neurodevelopmental disorder presenting with repetitive and restrictive patterns of behaviour, and persistent deficits in social communication across more than one context (American Psychiatric Association, 2013). Alongside deficits in social cognition there is evidence for difficulties in other cognitive processes, including executive functioning such as working memory, planning and cognitive and emotional regulation, as well anomalies in global processing with a tendency to focus more on detailed ‘local’ information within individuals with ASD as well as those with high autistic traits, a subclinical constellation of traits that is part of a broader autism phenotype (Cody et al., 2014; Richmond et al., 2013). Furthermore, comorbid diagnoses are prevalent in individuals with ASD, with sleep disturbance, anxiety and depression frequently also observed (Breslau, et al., 1996; Kerns et al., 2015; Liu et al., 2006). Currently, little is understood about how these varied comorbidities might contribute to or explain various cognitive difficulties in ASD.

Three prominent cognitive theories have been proposed to underpin ASD symptomologies. These theories seek to explain various features thought to be at the core of ASD, specifically with difficulties in social cognition explained by a deficit in theory of mind (ToM) (Gerlach and Starfelt, 2018; Jones et al., 2018; Premack & Woodruff, 1978); impairments in cognitive performance explained by the executive function (EF) deficit theory (Baron-Cohen et al., 1985; Cantio et al., 2016; Russell, 1997); and finally perceptual and cognitive biases towards detailed or local information processing has been explained by the weak central coherence (WCC) theory (Frith & Happé, 1994; Nuske & Bavin, 2011).

Premack and Woodruff (1978) defined ToM as the ability to impute mental states to oneself, and to understand that others have mental states that are different to one’s own. Baron-Cohen et al. (1985) further posited that individuals with ASD may have deficits in ToM after children with ASD did not employ ToM when tested on a belief question. The question involved guessing where ‘Sally’ would think the location of her marble might be after leaving it in a box, and after which it was secretly moved to another location. Individuals with ASD thought Sally would not go back to the original place where she left her marble, but would instead go to the current location, thus illustrating reduced capacity to understand Sally’s beliefs (Baron-Cohen et al., 1985). Individuals with ASD demonstrate persistent deficits in social cognition, evidenced through a reduced capacity for ToM in individuals from a young age (Baron-Cohen et al., 1985; Smith et al., 2010). There are also suggestions of a relationship between ToM, and restrictive and repetitive behaviours observed in individuals with ASD (Jones et al., 2018). A basic level of ToM involves the ability to recognise emotions and to attribute mental states from facial expressions, which has been demonstrated to be impaired in individuals with ASD (Andreou & Skrimpa, 2020; Baron-Cohen et al., 2001a, 2001b; Jones et al., 2018; Smith et al., 2010).

While ToM has been useful in furthering our understanding of communicative and social difficulties and to a lesser extent restrictive and repetitive behaviours in ASD (Baron-Cohen et al., 2001a, 2001b; Peterson, 2014), there also exist a number of difficulties that are unrelated to social patterns of behaviour. In fact, it is evident that individuals with ASD face deficits in executive functioning, including in planning, working memory, cognitive flexibility, self-regulation and response inhibition (Demetriou et al., 2018; Happe et al., 2006; Hill, 2004; Johnston et al., 2019; Russell, 1997; Storch et al., 2013; De Vries & Geurts, 2015). Importantly, working memory deficits are likely to influence key areas such as reading, attention, intelligence, and reasoning ability (Wang et al., 2018). Several working memory studies have demonstrated that individuals with ASD performed worse particularly on spatial working memory tasks compared to verbal or auditory working memory tasks (Baltruschat et al., 2011; Bodner et al., 2019; Habib et al., 2019; Wang et al., 2017).

Finally, the WCC theory seeks to explain the observed local processing bias and suggests that individuals with ASD have trouble integrating information but also show ability to process local details more competently at the expense of global meaning (Burnette et al., 2005; Frith & Happe, 1994; Frith & Frith, 2001; Happe & Frith, 2006). A number of studies have provided evidence that substantiates a preference for local processing by individuals with ASD showing that WCC can impact perceptual and visuospatial processing, restricted event schemas (cognitive tools for social understanding), comprehension and inferential processing of spoken narratives and ToM (Booth & Happe, 2018; Frith, 2003; Frith & Happé, 2006; Happe, 1999; Loth et al., 2008; Neufeld et al., 2020; Nuske & Bavin, 2011).

One difficulty with investigations into social, cognitive and perceptual anomalies in ASD, is the heterogenous nature of the disorder. This difficulty is compounded by the common comorbidities often associated with ASD (Mannion & Leader, 2013; Matson & Goldin, 2013). The presence of such comorbidities may impact the relationship between the behavioural traits of ASD and various aspects of social and cognitive functioning.

Sleep problems have been found in 50–80% of children with ASD compared to 9–50% in age-matched typically developed children (Kotagal & Broomall, 2012). Sleep problems typically include insomnia, parasomnias (nightmares, wakes screaming, enuresis), hyposomnias, sleep related breathing, and movement disorders (Richdale & Schreck, 2009). Individuals with ASD who have poor sleep have been found to have problems with attention span, social interactions, and with increased severity of autistic symptoms such as oppositional behaviour, aggression, explosiveness, attention deficit, impulsivity hyperactivity, anxiety, depression and mood variability (Deserno et al., 2019; Malhi et al., 2019; Mayes & Calhoun, 2009).

A study by Salmela et al. (2019) investigated the relationship between autistic traits and sleep by examining subclinical autistic traits in the general population, and demonstrated how elevated levels of autistic traits were associated with shorter weekday sleep duration, when comorbid psychiatric symptoms such as anxiety and depression were controlled for. The results suggested that subclinical autism traits should be considered as a possible antecedent to affecting sleep (Rzepecka et al., 2011; Salmela et al., 2019).

Anxiety is another highly prevalent comorbidity in ASD, with varying levels of prevalence being noted, ranging from 11 to 84% of individuals with ASD estimated to have an anxiety disorder (White et al., 2009). Such anxiety was shown to predict poor executive functions in adolescents with ASD (Hollocks et al., 2014). Indeed it has been suggested that anxiety moderates the relationship between autistic symptom severity and quality of life (Smith et al., 2019). Previous research has also demonstrated a relationship between anxiety and autism traits in the general population (Salmela et al., 2019). While there is little research on how these factors together impact cognitive performance, several studies have shown the negative impacts of anxiety on cognitive performance in neurotypical individuals (Gayete et al., 2020; Shilton et al., 2019). It has been posited that anxiety restricts the capacity of working memory (Shackman et al., 2006) and increases a visual perceptual bias towards local information (Basso et al., 1996; Shilton et al., 2019).

Along with sleep disturbance and anxiety, studies have also shown depression to be a common psychiatric comorbidity in ASD, with 70% of individuals having experienced at least one episode of major depression and 50% having had a recurrent depressive disorder (Bitsika & Sharpley, 2015; Leyfer et al., 2006; Sturm & Kasari, 2019). Again, although limited research exists on how depression impacts on cognitive performance in ASD, a number of studies show the negative impacts of depression on cognitive performance in individuals without ASD. For example, individuals with depressed symptoms demonstrate impairments in executive function, including working memory, possibly due to competition between attempts to direct attentional resources to the task and away from the distracting effects of automatic negative thoughts (Christopher & MacDonald, 2005).

Consideration of comorbid anxiety and depression may be further complicated by any reported gender and/or sex differences found in the autism spectrum (Sedgewick et al., 2020). In fact there is now increased awareness that the expression of autism is different for females than males (Rynkiewicz & Łucka, 2018). In those with ASD, there is some mixed evidence for gender differences in terms of psychopathology, though in adults it appears that women are more often diagnosed with comorbid anxiety and depression than men (see Napolitano et al., 2022 for a review). When examining autistic traits in the general population similar patterns appear evident, with higher rates of anxiety in female adults with higher autistic traits (Kanne et al., 2009). Furthermore, other aspects of cognition, including working memory have been suggested to have a different relationship with autistic features depending on gender (Chouinard et al., 2019). In the case of theory of mind, there are some reports females in the general population having superior theory of mind compared with males (Giovagnoli, 2019), though this difference was small and non-significant when using the Reading the Mind in the Eyes task (Baron-Cohen et al., 2001a, 2001b). Females compared to males with autism appear to show no differences in ToM, despite higher social reciprocity, indicating behavioural camouflaging (Livingston et al., 2019; Wood-Downie et al., 2021).

A wide-ranging literature has highlighted the complexity in understanding the social, cognitive and perceptual difficulties associated with ASD. For example, difficulties in working memory are associated with ASD and sleep (Calhoun et al., 2019) as well as anxiety and depression (MacNamara et al., 2019; Smith et al., 2018; Storch et al., 2013). In this regard, an understanding of EF deficits in ASD should take into account comorbid factors such as sleep, depression and anxiety. Similarly, there is some evidence that those with depression and anxiety have impairment in ToM (Burnette et al., 2005; Hezel & McNally, 2014; Washburn et al., 2016; Wolkenstein et al., 2011). In the neurotypical population, sleep deprivation has also been shown to impair judgement of facial emotions suggesting poor sleep may lead to impairment in the identification of social cues (Van der Helm et al., 2010).

Thus, the present study sought to examine the possibility that common comorbidities may mediate this relationship between ASD and core cognitive, social, and perceptual anomalies. As a first step to systematically control for the heterogeneity and common comorbidities in ASD, this study adopted the dimensional view of autism and examined autistic traits in the general population (Austin, 2005; Baron-Cohen et al., 2001a, 2001b; Landy & Chouinard, 2016; Laycock et al., 2014). This approach of researching the broader autism spectrum suggests that autism traits are normally distributed throughout the general population, with those with a diagnosed ASD forming one end of this spectrum. A number of self-report surveys are now commonly used to measure quantitative autism traits in the non-clinical population (Baron-Cohen et al., 2001a, 2001b; Hurley et al., 2007; Kanne et al., 2012), often referred to as the broader autism phenotype (Hurley et al., 2007). A large body of research highlights how neurotypical individuals with higher autism traits, but who do not meet criteria for an ASD diagnosis, are associated with similar comorbid psychiatric issues (Kanne et al., 2009), reduced social cognition (Poljac et al., 2013; Wallace et al., 2010), reduced executive function (Christ et al., 2010), and enhanced local-focussed perceptual processing (Cribb et al., 2016), as those with a diagnosed ASD. This dimensional approach thus provides research opportunities with larger sampling pools, control over other confounding variables such as intellectual functioning and offers higher practicality for ASD studies (Hyseni et al., 2019; Landry & Chouinard, 2016).

Specifically, this study aims to explore the relationship between autistic traits and (1) working memory performance, (2) mental state attribution from faces, and (3) global and local visual information processing, in an attempt to represent elements of three prominent theories of autistic impairment, namely executive function deficits, Theory of Mind deficits, and Weak Central Coherence, respectively. The impact of autistic traits on these cognitive variables will be examined by also testing the mediating roles of sleep disturbance, anxiety, and depression. To our knowledge no previous study in the field has explored this integral modelling in a single study design, and thus this empirical investigation should benefit research and practical intervention initiatives.

## Method

### Participants

From 243 adult participants recruited via social media (*n* = 39) and an international crowd sourcing platform (MicroWorkers; *n* = 204; Shapiro et al., 2013; Tompkins, 2019), 188 complete datasets from the general population were attained over a period of approximately 1 month. Demographic and descriptive information is provided in Table 1. Participants from Facebook were offered the option to enter a draw to win a $50AUD voucher, while MicroWorkers participants received a credit worth 2USD (Bennetts et al., 2019; Cahill et al., 2019).

**Table 1:** Demographics and descriptive statistics of participants (*N* = 188)

Item	Total (frequency)
Mean age years (*SD*)	30.49 (*8.75*)
Gender	
Male	108 (57%)
Female	78 (41%)
Other^a^	2 (2%)
Education attainment	
High school or below	51 (27%)
Studying at university	36 (19%)
Completed university	77 (41%)
Masters or higher	24 (13%)
Country of residence	
Australia	30 (16%)
Canada	14 (8%)
New Zealand	4 (2%)
United Kingdom	26 (14%)
United States	111 (60%)
Other	1 (.5%)
History of mental health^b^: diagnosed	137 (74%)
Autism spectrum diagnoses Participant diagnosed	16 (9%)
Family diagnosed	17 (9%)
Sleep disorder diagnoses^c^: diagnosed	15 (8%)

^a^Due to the small number of the other gender, these two cases were treated as missing gender in the later analysis to prevent statistical instability

^b^Mental health diagnoses: ADHD, anxiety, anxiety/depression, anxiety/autism, borderline personality disorder, depression/PTSD, panic disorder/PTSD/depression, schizotypal, social anxiety disorder, obsessive compulsive, general anxiety disorder/social phobia, and non-diagnosed

^C^Sleep narcolepsy, sleep apnoea, obstructive sleep disorder, insomnia/bruxism, insomnia, historical sleep maintenance disorder

### Materials

#### Demographic Information

All participants were required to indicate their age, gender, level of education, handedness, and a brief history of health including mental health, sleep disorders and a question about personal and family history of having an ASD diagnosis.

#### Autism Spectrum Quotient (AQ)

The AQ is a 50-item questionnaire that assesses quantitative autistic traits in the general non-clinical population. Participants indicated their level of agreement with statements relating to domains of social skills, communication, imagination, attention to detail, and attention switching (Baron-Cohen et al., 2001a, 2001b). Responses to a four-point Likert scale was scored using a binary scoring system as described by Baron-Cohen et al., (2001a, 2001b). Answers that endorsed the autistic trait scored one point. The reliability of the total AQ score was α = .70 in this sample.

#### Depression Anxiety Stress Scale (DASS-21)

The DASS-21 was used to assess depression and anxiety symptoms experienced over the past week (Lovibond & Lovibond, 1995). The DASS-21 consists of 21 questions using a 4-point Likert scale ranging from ‘did not apply to me at all’ (0) to ‘applied to me very much or most of the time’ (3), such as “I couldn’t seem to experience any positive feeling at all”. The reliability of the DASS–21 was α = .93 for Depression and was α = .82 for Anxiety in this sample.

#### Pittsburgh Sleep Quality Index (PSQI)

The PSQI questionnaire was used to assess sleep quality and quantity (Buysse et al., 1989). It contains 19 items in the seven component domains of subjective sleep quality, sleep latency, sleep duration, habitual sleep efficiency, sleep disturbance, use of sleep medication and daytime dysfunction. Participants completed a four-point Likert scale that ranges from very good (0) to very bad (3). The component scores were combined to produce the Global Sleep Quality Score of between 0 and 27, with participants scoring 6 or greater considered to be poor sleepers. The reliability of the PSQI was α = .72 in this sample.

#### Corsi Block Tapping Task

**Fig. 1:**
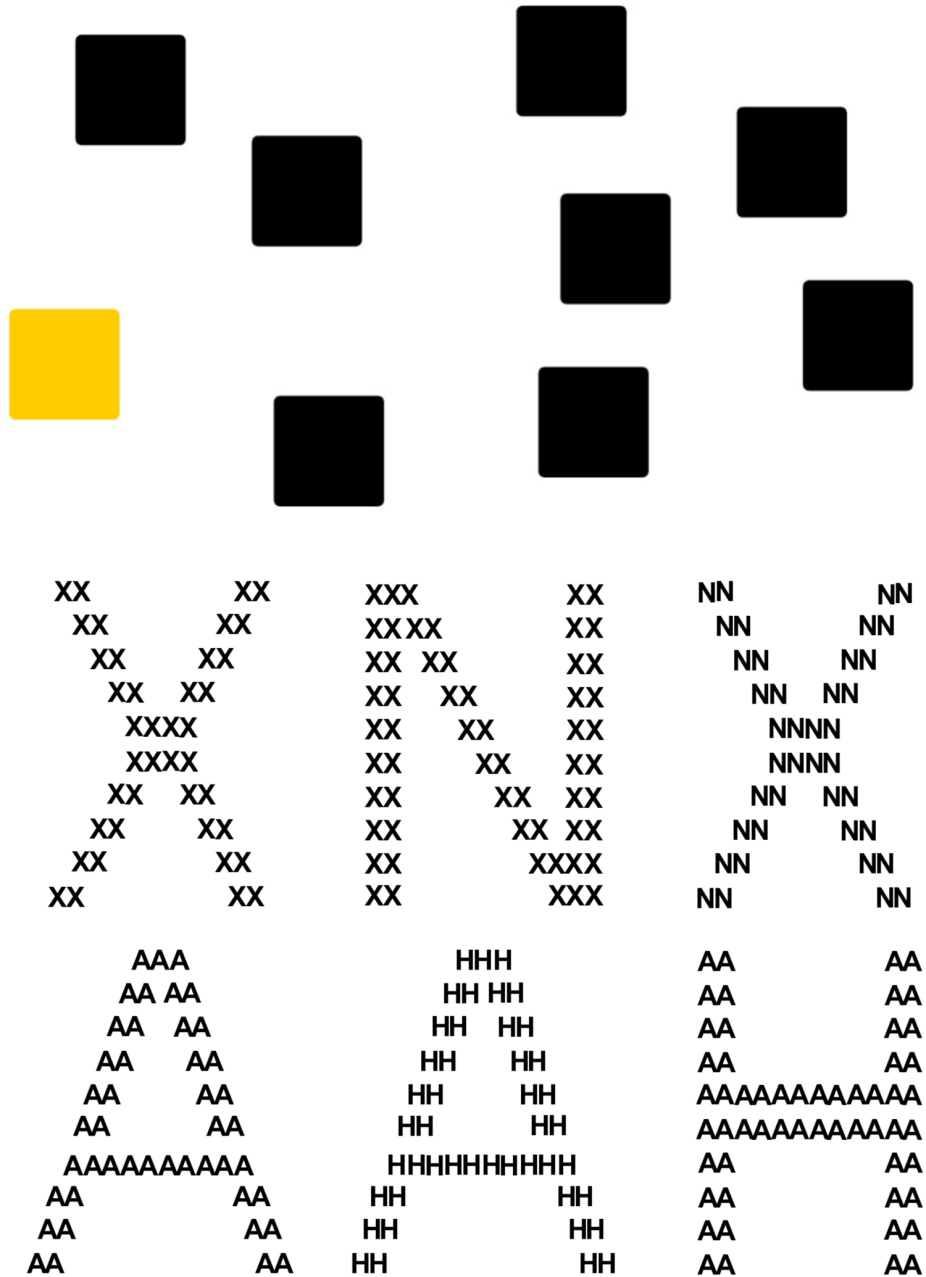
Top: Illustration of the online Corsi block tapping task presented to participants. Following a sequence of blocks that flashed a different colour, participants used a computer mouse to press the same sequence of blocks. Bottom: Stimuli used in the Navon Task: From left to right, Congruent, Global Incongruent, and Local Incongruent Stimuli for X (upper) and A (lower) target stimuli. Participants indicated whether there was an X or A present, which could be at either the global or local levels

For an index variable of Executive Function, the Corsi Block task was used to assess visuospatial working memory performance (Vandierendonck et al., 2004). An online version was developed for this study using the task builder provided in the Gorilla software. A similar digital version of this task has been successfully utilised previously (Brunetti et al., 2014). As Fig. 1 (top panel) illustrates, the screen presented participants with 9 black squares (‘blocks’) scattered on a white background. One at a time a single block changed to the colour blue for 500 ms before returning to the original black colour. Following a 250 ms interval, another block changed colour. Initially a two-block sequence was shown and participants were required to use the mouse to click on the same sequence of blocks previously presented. The sequence span increased after two repetitions regardless of participant accuracy until a sequence of 8 was presented. Performance was scored as the number of the blocks with the longest span correctly recalled (Brunetti et al., 2014). The Corsi Block Tapping task has been previously reported to have moderate test-rest reliability (White et al., 2018; Williams et al., 2005).

#### Navon Task

For an index variable for Weak Central Coherence (WCC), the Navon task was used to assess a global precedence which refers to a bias towards cognitive processing the overall structure of a stimulus over its individual elements (Hayward et al., 2018; Navon, 1977). As illustrated in Fig. 1 (bottom panel), participants were presented with a central large global letter (H, N, A or X) which was created from the arrangement of smaller local letters (H, N, A or X). The small letters could be either congruent or incongruent with the large letters.

Participants were required to respond by a keyboard press whether an A or an X was present at either local or global level. Thus, on any trial there could be an A or X at the global but not the local level (global incongruent), or an A or X at the local but not the global level (local incongruent), or an A or X at both global and local levels (congruent) with 12 trials for each condition resulting in 36 trials. Reaction time for correct trials were analysed, and the mean reaction time score for each of the three conditions (global incongruent, local incongruent, and congruent) was calculated for each participant.

For statistical analysis, three final outcome variables were computed: (1) local interference (reaction time for global incongruent stimuli—reaction time for congruent stimuli) indicating the interference of task irrelevant incongruent local stimuli on global processing (2) global interference (reaction time for local incongruent stimuli—reaction time for congruent stimuli) indicating the interference of task irrelevant incongruent global stimuli on local processing (3) global precedence (reaction time for local incongruent stimuli—reaction time for global incongruent stimuli) for which a positive score indicates faster reaction time for global than local stimuli, whereas a negative score indicates faster reaction time local than global stimuli. The test re-rest reliability for a Navon task was recently reported to have an IntraClass Correlation (ICC) of .74 (Hedge et al., 2018).

#### Reading the Mind in the Eye Test

For an index variable of ToM, the revised version of the Eyes test was used to assess individual differences in social cognition (Baron-Cohen et al., 1997, 2001a, 2001b). The test included 36 photographs of faces showing only a rectangle area around the eyes with four mental state terms (e.g., playfulness, desire, worrying, cautiousness). Participants were required to select the corresponding description that best matched the expressed mental state in the photograph. Performance was determined by the sum total of correct responses. The reliability index for this study sample was Cronbach’s α = .83.

### Procedure

In accordance with the university research ethics approval, participants completed the questionnaires and cognitive tasks online using the Gorilla Experiment platform (www.gorilla.sc) (Anwyl-Irvine et al., 2019). Participants provided online consent and completed a brief demographics survey. Next the three measures were completed in a randomized order, and subsequently the three cognitive tasks which were also randomised. Randomization used a Latin square design. The study was completed in 30 min on average.

### Data Analysis Overview

Statistical Package for the Social Sciences (SPSS), version 26 was used for all analyses. For the Navon task, participants with reaction time data faster than 200 ms or slower than 1500 ms on any congruence condition were treated as ingenuine responses and excluded from all analyses. This resulted in 43 cases excluded from the main data set. Outliers of the Navon task and Corsi Block task variables were also excluded while there were no outliers on the Eyes Test.

Autistic trait groups were established following a tertile split based on AQ scores from the current sample. This resulted in lowest tertile (AQ total score < 20, *n* = 69, 37%) and the highest tertile (AQ total score > 24, *n* = 62, 33%) constituting low and high AQ groups, respectively. For the main analysis the low AQ group had a mean AQ = 14.42 (*SD* = 3.15), and the high AQ group had a mean AQ = 29.27 (*SD* = 4.82). Thus while the AQ profile of our overall sample (*M* = 21.69, *SD* = 7.10) is higher than that reported in large population studies (e.g., Stevenson & Hart, 2017; *M* = 18.04, *SD* = 5.86), both AQ groups in the current study are significantly different from the sample mean in Stevenson et al. (*p*’s < .001), indicating good separation for high and low autistic tendency from the general population.

An analysis of variance (ANOVA) was conducted to test the mean differences between the high and low AQ groups for the PSQI, Depression, Anxiety, Corsi Block task, and the Eyes Test scores. For the Navon ANOVA the reduced sample was analysed (low AQ, *n* = 59; high AQ, *n* = 43).

Multiple regression analysis was also conducted with the total AQ scores as the explanatory variable using the total sample to examine the AQ impact on comorbidity symptoms and cognitive performance, while controlling for demographics, diagnosis of ASD in the participants and family members, other mental health comorbid diagnoses, and sleep disorders.

Finally, Structural Equation Modelling (SEM) with AMOS (Analysis of a Moment Structures) version 26 was used to test multiple mediation roles of sleep disturbance, anxiety and depression in explaining the impact of autistic traits upon cognitive performance measured with Corsi Block task, Navon task, and the Eyes Test. The direct effect and indirect effects through the mediators were tested for their significance (Bentler, 2007; Bentler & Yuan, 1999; Mackinnon, 2012; Rigdon, 1996). Given the potential for gender to influence these effects, gender was also included in the SEM analysis.

## Results

### Descriptive Statistics and Pearson Correlations

After data cleaning and excluding outliers, descriptive statistics and Pearson’s correlations were tested between AQ score and the three comorbidity variables of sleep disturbance, anxiety and depression, and the cognitive test variables of Eyes Test, CBBT, Navon task (global interference, local interference and global precedence, Table 2). The results of the Pearson correlations indicated that there were significant positive correlations observed between AQ score and depression, anxiety and sleep, while from the cognitive measures, significant negative correlations were observed between AQ score and the Eyes Test and the global precedence measure from the Navon task. Further advanced main analysis was therefore conducted to examine underlying relationships between these variables.

**Table 2:** Pearson’s correlations between the autism quotient and study variables

		1	2	3	4	5	6	7	8	9
1	Autism quotient	–								
2	Depression	.46***	–							
3	Anxiety	.46***	.67***	–						
4	Sleep disturbance	.18*	.42***	.40***	–					
5	Eyes in the mind	− .22**	− .06	− .21**	− .03	–				
6	Corsi block task	− .12	− .09	− .15*	− .14	.32***	–			
7	Global interference	− .13	− .12	− .05	.06	.05	− .08	–		
8	Local interference	.13	− .02	− .01	− .06	− .09	.03	.23**	–	
9	Global precedence	− .21	− .08	− .04	.10	.11	− .09	.62***	− .62***	–
	Mean	21.69	7.89	6.08	5.01	23.16	8.15	247.13	178.71	68.42
	Standard deviation	7.09	5.64	4.12	2.59	6.30	2.30	145.95	145.95	181.55

N = 188, except correlations involving Navon conditions, *n* = 144

***p < .001, **p < .01, *p < .05

### ANOVA and Multiple Regression Models: Differences Between Low and High AQ Groups

For the Corsi Block task, outliers with two or less than two correct responses were excluded, resulting in one participant from the low AQ group with only 1 correct response being excluded (representing > 3 SD from the group mean). ANOVA showed significant differences between the high and low autism trait groups for the Eyes Test, Corsi Block task, as well as for depression, anxiety, though not for sleep disturbance (Table 3).

**Table 3:** ANOVA results comparing high and low AQ groups, and regression analysis of outcome variables explained by AQ scores

Dependent variables	ANOVA (high vs low AQ)				Regression^a^ (AQ total scores)			
	*F*	*p*	η_p_^2^	Power	β	*p*	*R*^2^	*b* [LCI,UCI]
Mind in the eyes	**14.24 (1,129)**	** < .001**	**.10**	.96	**− .17**	**.02**	**.02**	− .15 [− .30, .01]
Corsi block task	**5.38 (1,125)**	**.02**	**.04**	.63	− .03	.17	.01	− .09 [− .27, .06]
Global interference	2.18 (1,100)	.14	.02	.31	− .16	.08	.07	− 3.16 [− 6.70, 0.38]
Local interference	2.76 (1,100)	.10	.03	.38	.09	.34	.04	1.75 [− 1.86, 5.36]
Global precedence	**6.79 (1,100)**	**.01**	**.06**	.73	**− .20**	**.03**	**.11**	− 4.91 [− 9.26, − 0.56]
Depression	**27.40 (1,129)**	** < .001**	**.18**	0.99	**.30**	**.00**	**.20**	.38 [.19, .41]
Anxiety	**35.10 (1,129)**	** < .001**	**.21**	1.00	**.36**	**.00**	**.27**	.22 [.14, .29]
Sleep disturbance	0.43 (1,129)	.52	.003	.10	.12	.17	.12	.04 [− .02, .09]

Significant results are in bold

^a^Covariates were controlled in the regression models and they included age, gender, education, mental health diagnosis, sleep disorder, ASD diagnosis for the participant and family members; *LCI* lower confidence intervals; *UCI* upper confidence intervals

**Fig. 2:**
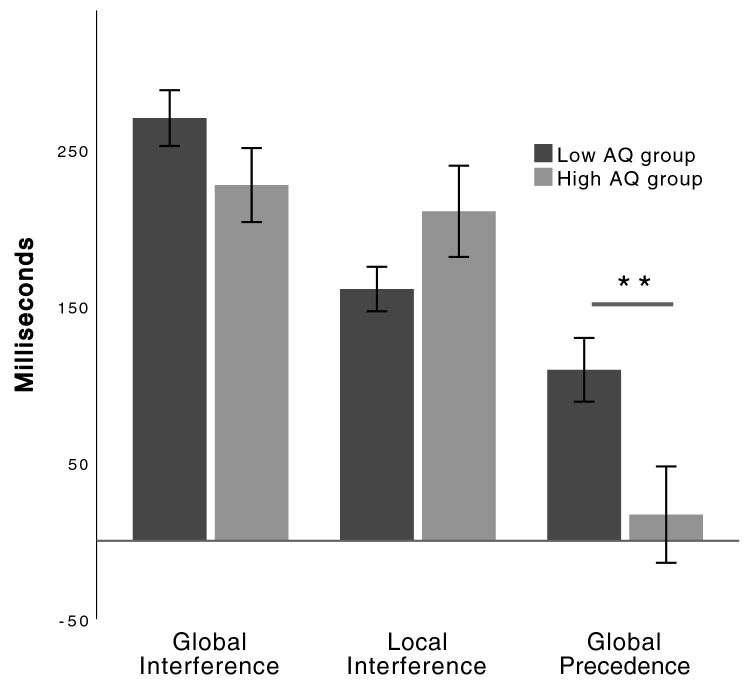
Three outcome variables for the navon task for high and low AQ groups. Global interference represents the difference in reaction time between congruent navon stimuli and incongruent stimuli with the target present at the local level. Local interference represents the difference in reaction time between congruent navon stimuli and incongruent stimuli with the target present at the global level. Global precedence represents the difference in reaction time between target identification for global and local incongruent stimuli. The high AQ group demonstrated significantly lower global precedence when compared with the low AQ group. Error bars indicate Standard Error of the Mean. **p < .01

For the Navon task, as seen in Fig. 2, the pattern of results was as expected with the low group showing more global interference on local processing, and less local interference on global processing than the high AQ group. However ANOVA showed no evidence for significant group differences on these measures (Table 3). To further examine this pattern of results, a mixed design ANOVA with Interference Level (Global vs Local interference) as the within subject factor, and AQ Group (high vs low) as the between subject factor was conducted. This analysis demonstrated a main effect of interference level [F(1,100) = 12.60, *p* = .001], no significant main effect of group [F(1,100) = 0.02, *p* = .884], and a significant interaction between interference level and group [F(1,100) = 6.79, *p* = .011]. Simple main effects were used to interpret this interaction, suggesting that while the low AQ group had significantly larger global than local interference (*p* < .001), the high AQ group did not show this difference (*p* = .536). When examining global precedence, a significant group difference was apparent indicating a larger global processing advantage in the low AQ group, when compared with the high AQ group (*p* < .01; see Fig. 2).

When participant age and gender, along with diagnosis of ASD and other comorbid disorders were controlled in multiple regression, the AQ total scores significantly explained only the Eyes Test, global precedence, depression, and anxiety (see Table 3).

### SEM: Mediation of Comorbidities in the Impact of AQ on Cognitive Performance

**Fig. 3:**
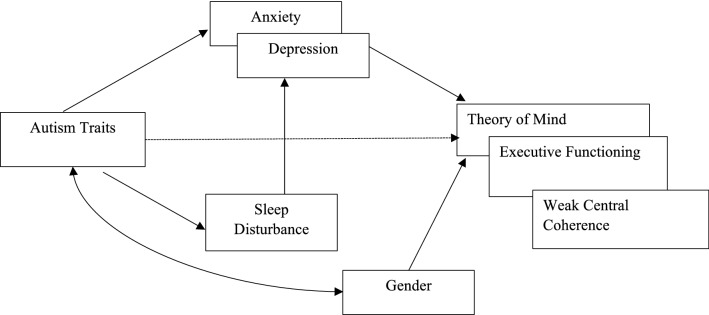
Final mediation SEM: the mediation effects of sleep disturbance, depression, and anxiety in the impact of autism traits on theory of mind, executive functioning and weak central coherence. Note. Error terms and correlations are omitted for simplicity

In exploring mediation roles among the comorbidity variables upon each of the cognitive performance variables, a series of multiple mediation SEMs were tested. As presented in Fig. 3, the most parsimonious model included multiple mediation paths (1) from autism traits to cognitive performance outcome variables via sleep disturbance and in turn through anxiety or depression, (2) from autism traits to cognitive performance outcome variables via anxiety or depression. In total, 10 models (= 2 s mediators (Depression or Anxiety) × 5 dependent variables (Eyes, Corsi Block task, three Navon variables) were tested. Model fit was analysed with χ^2^ to test for any significant differences in the covariance-variance matrices between the data and the model. Non-significant χ^2^ indicates good fit, and overall, all models demonstrated good fit with the data as shown in Table 4. As a second step, the individual paths tested in the models are detailed in the following.

**Table 4:** Mediation models: fit indices and proportions of explained variances in cognitive performance variables

Model path in explaining 5 dependent variables All models also included a path from autism traits to anxiety/depression	Model fit χ^2^	RMSEA	CFI	TLI	*R*^*2*^
*Model 1* Autism traits*→* sleep disturbance*→* anxiety*→* eyes in the mind	1.43	.00 CI .000, .093	1.00	1.10	.13
*Model 2* Autism traits*→* sleep disturbance *→* depression*→* eyes in the mind	.57	.00 CI .000, .052	1.00	1.10	.13
*Model 3* Autism traits *→* sleep disturbance *→* anxiety *→* Corsi block task	.20	.00 CI .000, .000	1.00	1.13	.06
*Model 4* Autism traits *→* sleep disturbance *→* depression*→* Corsi block task	.35	.00 CI .000, .000	1.00	1.13	.04
*Model 5* Autism traits *→* sleep disturbance *→* anxiety*→* global interference	3.41	.03 CI .000, .152	1.00	.97	.01
*Model 6* Autism traits*→* sleep disturbance *→* depression*→* global interference	2.00	.00 CI .000, .124	1.00	1.08	.01
*Model 7* Autism traits*→* sleep disturbance *→* anxiety*→* local interference	3.17	.02 CI .000, .145	1.00	.99	.04
*Model 8* Autism traits*→* sleep disturbance *→* depression*→* local interference	1.06	.00 CI .000, .092	1.00	1.15	.03
*Model 9* Autism traits*→* sleep disturbance *→* anxiety *→* global precedence	1.82	.00 CI .000, .119	1.00	1.07	.05
*Model 10* Autism traits*→* sleep disturbance *→* depression*→* global precedence	1.04	.02 CI .000, .092	1.00	1.14	.05

Non-significant χ^2^ indicates the models fit the data supporting the null hypothesis posing no significant difference in the covariance-variance matrices between the data and the model. *df* = 3. RMSEA: root mean square error of approximation, CFI: Comparative Fit Index, TLI: Tucker–Lewis Index, CI: 95% Confidence Interval. Sample sizes: eyes in the mind task (*N* = 186), Corsi block task (*N* = 177), global incongruence (*N* = 137), local incongruence (*N* = 142), global Precedence (*N* = 139)

**Fig. 4:**
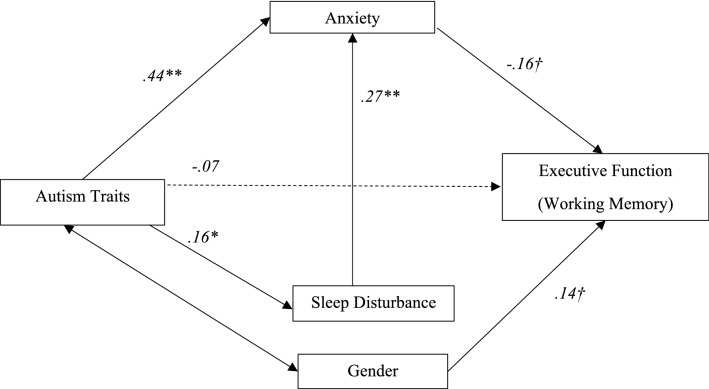
Standardised SEM coefficients: the mediation effects of sleep disturbance and anxiety in the impact of autism traits on executive functioning. *Note*. For the model fit, refer to Table 4. Sleep Disturbance: Good Sleep = 0, Poor Sleep = 1, Gender: male = 1, female = 2, ^*†*^*p* < .10, *p < .05, **p < .01, Error terms, correlations, and related coefficients are omitted for simplicity. Solid lines depict significant paths while dotted lines depict non-significant effects

In none of the models tested were all paths found to have significant relationships. One model however was suggestive of illuminating important relationships between the variables. Figure 4 presents the SEM coefficients and paths of the mediation model on working memory, measured with the Corsi Block task. All paths within this model were significant, except for the final path. Although statistically non-significant at the conventional alpha level (.05), there was the suggestion of full mediation effects of sleep and anxiety: autism traits explained the variance in working memory performance but only indirectly through sleep disturbance and anxiety. Specifically, autism traits significantly predicted sleep disturbance (*p* < .01) which in turn significantly explained anxiety (*p* < .01), though anxiety was associated with working memory performance with a non-significant relationship (*p* = .06). As detailed in Table 5, a one-unit increase in autism traits was associated with a 0.44-*SD* increase in anxiety and a 0.16 − *SD* increase in sleep disturbance which in turn indirectly decreased working memory performance with − 0.07 *SD* and − 0.01 *SD* respectively.

**Table 5:** SEM coefficients of the final model: mediation of anxiety and sleep disturbance in the impact of autism traits on working memory

	Coefficients			
Predictors and paths	*b*	*SE*	*β*	*p*
Autism traits → sleep disturbance	0.012*	.005	.16	.029
Sleep disturbance → anxiety	2.303**	.534	.27	.000
Autism traits → anxiety	0.267**	.038	.44	.000
Anxiety → EF	− 0.081*†*	.043	− .16	.060
Autism traits → EF	− 0.020	.026	− .07	.437
Gender → EF	.59*†*	.318	.14	.062
Autism traits→ anxiety→ EF	− .02*†*	.01	− .07	.101
Autism traits→ sleep disturbance→ anxiety	.04*	.02	.07	.019
Autism traits → sleep disturbance→ anxiety→ EF	− .003	.003	− .01	.199

*p < .05, **p < .01, *†p* < .10, β: Standardised coefficient, *b:* unstandardized coefficient, SE: standard error. EF: executive functioning indexed by the working memory variable, Corsi Block Task total scores. Gender: male = 1, female = 2

The full mediation of sleep and anxiety was not observed either when examining the measures of ToM and WCC. Autistic traits directly explained local interference (*b* = .19, *p* = .044) without mediation of sleep disturbance and anxiety or depression in the models. Mental state attribution, the Eyes test scores (*b* = − .14, *p* = 0.073) and global precedence (*b* = .04, *p* = 0.064) demonstrated trends for being explained by autistic traits, but were not statistically significant. In contrast, global interference (*b* = − .03, *p* = .780) was not explained directly by autistic traits and any mediation of sleep disturbance and anxiety or depression was not significant in the models either.

## Discussion

The present study investigated potential mediations of prevalent ASD comorbidities including sleep disturbance, anxiety and depression in the relationship between autism traits and cognitive performance using an international community sample from the general population. The dimensional approach to exploring the broader autism phenotype was used to maximise the research capacity with a large sample size and sophisticated statistical modelling. Results demonstrated that autism traits were linked to cognitive measures, and also to common comorbidities, though as will be discussed below, performance on a measure of working memory was mediated by both sleep and anxiety.

### Autism Traits: Consistency and Complexities in Comorbid Symptoms and Cognitive Performance

Individuals with low and high autistic traits in the sample showed significant differences in mental state attribution as a ToM index; in executive function as measured by a spatial working memory task; and from a Navon global/local task when assessing global precedence, though not global or local interference, which were taken as measures of Weak Central Coherence. In addition, significant autistic trait group differences were established for anxiety and depression, while no group differences were found for sleep disturbance. These findings in general are supportive of previous findings of impairments in the autism spectrum in a range of social, cognitive and perceptual tasks (Baltruschat et al., 2011; Baron-Cohen et al., 1985; Plaisted et al., 1999; Smith et al., 2010; Wang et al., 2018), as well as difficulties in mental health (Leyfer et al., 2006). We did not replicate the previous finding of abnormalities in sleep linked to autism traits in the general population (Salmela et al., 2019). Importantly however, Salmela et al. showed an association between autisic traits and objective measure of sleep using wrist actigraphy recordings, whereas similar to the current findings these authors found no association between autism traits and self-reported sleep quality (also using the PSQI). These findings together converge with evidence that similar brain differences are found in high autism trait groups as in clinical populations with ASD (Focquaert & Vanneste, 2015) and are supportive of research utilising this dimensional approach to understand autistic traits, as a complement to clinical ASD studies (Landry & Chouinard, 2016).

Regarding the Navon task which was used as a measure of global/local processing and the WCC theory, results indicate significant differences for global precedence indicating that those with higher autism traits were slower to respond to global stimuli relative to local stimuli suggestive of a reduced bias for global processing. On the other hand the results established no group differences in local or global interference, while it should be noted that the group means were in the expected direction, with high AQ participants having greater local interference on global processing that the low AQ participants. The lack of significance on the local interference and global interference measures may be attributable to the online testing environment where reduced signal to noise is expected, particularly for a reaction time measure. Another complication in comparing Navon task results from different studies is the wide range of stimulus designs (e.g., size of local to global stimulus ratios, letters vs numbers vs arrows) (Hayward et al., 2018; Navon, 1977). Nevertheless, as can be seen in Fig. 2, and as suggested by a second analysis, low AQ participants demonstrated more global interference than they did local interference, whereas the degree of global and local interference was equivalent in the high AQ group, again suggested of a reduced global bias. These results thus broadly support the notion that autistic traits, similar to ASD findings (Plaisted et al., 1999) are associated with a bias against processing global information.

Notably, only mental state attribution, global precedence, depression, and anxiety were significantly explained by autistic traits in hierarchical regression models when the total AQ scores were modelled using the whole sample with demographic variables including presence of a mental disorder and ASD diagnoses controlled. These results, with the measure of working memory (Corsi block task) not significant, suggest more complex relationships between the variables which called for the subsequent SEM modelling with more sophisticated mediation analysis. While limited utility of using the AQ scores has been observed in the regression results, it is also suggested that the covariates of age, gender, education, mental health including ASD, and sleep disorders should be taken into account in future research designs (Ruzich et al., 2015).

### Impacts of Comorbidities: Mediation Roles for explaining Working Memory?

For the main investigation, the SEM results demonstrated that although statistically non-significant, sleep disturbance and anxiety were suggested to fully mediate the impact of autism traits on working memory as an index of executive function. Specifically, SEM showed autism traits explained the variance in working memory measured with the Corsi Block task, but only indirectly through sleep disturbance and anxiety, suggesting a full mediation. Importantly, this finding did not reach statistical significance, likely due to the the large number of predictors variables included in the analysis. The large number of estimated coefficients overburdened the models with the relatively small sample size for the complexity of the multiple mediation paths. In understanding the convoluted nature of the interactions of neurological, psychological, and behavioural factors and symptoms in autism, even larger samples would be required to confirm these relationships. Furthermore, replication in clinical samples will also be important to confirm the relevance to those diagnosed with ASD. Interestingly, this mediation was not observed for depression. The suggestion of anxiety and sleep mediating the relationship between autism traits and executive function is consistent with recent research into the relationships between ASD and sleep with elevated levels of subclinical autistic traits shown to be associated with shorter weekday sleep duration, when comorbid psychiatric symptoms such as anxiety and depression were controlled for (Salmela et al., 2019). While the present study supported Salmela et al. (2019), where subclinical autism traits should be considered as a possible antecedent to affecting adolescent sleep, we now also suggest that anxiety was further explained by sleep disturbance and in turn impacted working memory performance.

Previous research has highlighted that in children with ASD, autism traits were positively associated with chronic stress hormone level (assessed from cortisol in hair), while in the same children cortisol was negatively associated with spatial working memory performance (Ogawa et al., 2017). Interestingly, Ogawa et al. (2017) found no similar relationships between cortisol, autism traits and a revised child-version of the Eyes task, which also seems to support the results of the present study. The maintenance of visuospatial information in working memory is understood to be reliant on a right-lateralised fronto-parietal network (Rottschy et al., 2012). Atypical development of structural connectivity within the frontoparietal network has been demonstrated in ASD (Lin et al., 2019), with a recent meta-analysis pointing to the importance of this network for underlying executive dysfunction in ASD (Ma et al., 2015; May & Kana, 2020).

Anxiety and sleep difficulties are both associated with impairments in cognitive functioning, including in working memory (Dai et al., 2020; Shackman et al., 2006). Similarly, while both anxiety and sleep deprivation have detrimental effects on frontoparietal networks (Ma et al., 2015, 2019), previous research have highlighted how individuals with ASD and sleep problems are more likely to suffer from anxiety and have difficulties with a number of cognitive and behavioural domains (Malhi et al., 2019; Mayes & Calhoun, 2009; Mughal et al., 2020). The current results thus build on these findings by suggesting that severity of autistic behaviours may predict anxiety, and in turn sleep difficulties, which finally may mediate working memory performance. A recent meta-analysis found broad difficulties in executive function in ASD compared with neurotypical control participants, though it was suggested that this may not achieve clinical sensitivity (Demetriou et al., 2018). However, our findings indicate that future research may need to investigate the causal influence of anxiety and sleep difficulties on executive functioning, to improve the understanding of the relationship between autistic behaviours and executive functioning.

Calhoun et al. (2020) have recently shown that in adolescents with ASD, those who had a sleep disturbance demonstrated a relationship between working memory and learning problems, while those without sleep disturbance did not show this relationship. The authors suggest that interventions may need to target both sleep and working memory. We suggest that anxiety may also be a critical target of intervention to allow improvement in both sleep and working memory, though future studies will need to confirm the nature of these mediating relationships in clinical populations.

Contrary to our hypotheses, sleep disturbance, anxiety and depression did not mediate the relationship between autism traits and performance of ToM (mental state attribution) and WCC (Navon task on global and local processing) measures. However, although both anxiety and ASD have been associated with a disturbance in ToM, there is some evidence that the nature of this disturbance may differ between conditions. For example it has been suggested that while those with ASD fail to use Theory of Mind to infer another’s mental state, anxiety has been associated with ‘over-mentalising’ (Washburn et al., 2016), in which mental states are inferred, though they are incorrectly made. This difference in ToM abilities, while anomalous in both clinical conditions, would clearly be expected to complicate the relationship between autism traits, anxiety and ToM.

The failure to establish mediating relationships in predicting measures of ToM and WCC may also be due to the online cognitive task settings and limited refinement in the stimuli. In particular the Navon task requires accurate measurement of motor reaction time which may be expected to have increased inter- and intra-participant variability. Although online cognitive testing has begun to be more common, with some recent validation studies (Wesnes et al., 2017), there are still technical difficulties in ensuring consistent conditions for all participants. More controlled laboratory studies with rigorous sampling should cross-validate the results of the present study.

### Limitations and Future Research and Practice

One of the limitations in this study was that the PSQI questionnaire results may have been impacted due to an error in omission of a single item and subsequent default scoring of 0 for those responses. Due to the non-linear nature of scoring for the PSQI, this is not suggested to have altered the ability of this scale to detect sleep disturbance. Nevertheless, some caution is required in comparing PSQI scores to previous studies, and may have influenced the current findings on sleep disturbance in subtle ways. In addition further larger sample sizes will allow more comprehensive mediation models with all relevant covariates to be controlled for which was not available for the present study. Due to the limited scope of this study, subscales of AQ such as social skills, attention shift, and communication skills were not analysed which should be examined in future work. Similarly, sub factor analysis of sleep disturbance such as ‘Getting to Sleep Difficulty’ and ‘Activity and Enthusiasm Problem’ by providing more precise unidimensional measures may provide more clinically meaningful elaboration of the relationship between variables. Finally, it is important to caution that due to the sample being a non-diagnosed community sample, clinical implications may be different for diagnosed persons. It will be important to cross-validate these findings in a clinical population.

### Conclusion

This study examined three major cognitive theories to understand the autistic symptoms with three common comorbidities. It is the first time where all three ASD theories have been tested in a single study design to assess how prominent comorbidities may impact the key cognitive symptomologies of ASD. In terms of methodology, the present study also demonstrated time and cost efficiencies through using an online experimental platform and benefits from using sub-clinical autism traits assessed through the AQ with heightened approachability, larger sampling pools, and higher practicality for ASD research.

While no significant mediation effects were found regarding the tasks expected to test WCC theory and ToM, the present results demonstrated that the comorbidities of sleep disturbance and anxiety are likely to play a mediating role in the impact of autistic traits on working memory. This finding thus adds evidence to the importance of EF theory in explaining that the deficits in executive functioning in autism may be indirect via the mediations of sleep disturbance and anxiety that were impacted by autistic traits. This suggests that treating the symptoms of sleep disturbance and anxiety may lead to improvements in working memory and possibly in other executive functions (Andersen et al., 2008; Baltruschat et al., 2011; Storch et al., 2013). Future research with different measures of executive function such as emotion regulation may expand the present findings, and determine how specific the mediating role of anxiety and sleep is to visuospatial working memory.
